# Congenital Methemoglobinemia and Unstable Hemoglobin Variant in a Child With Cyanosis

**DOI:** 10.7759/cureus.16081

**Published:** 2021-07-01

**Authors:** Mohammed Alsabri, Kusum Viswanathan, Anthony Elias, Mario Peichev

**Affiliations:** 1 Pediatrics, Brookdale University Hospital Medical Center, Brooklyn, USA; 2 Pediatrics, New York Institute of Technology (NYIT) College of Osteopathic Medicine, New York, USA; 3 Pediatric Hematology and Oncology, Brookdale University Hospital Medical Center, Brooklyn, USA

**Keywords:** methemoglobinemia, cyanosis, hemoglobinopathy, congenital, pediatrics

## Abstract

Methemoglobinemia (Meth) is a rare hemoglobin (Hb) disorder with distinguished clinical features and complex pathophysiology. We present a three-year-old female who was diagnosed with congenital methemoglobinemia when she presented with peri-oral cyanosis and profound oxygen desaturation in the 20-30% range. This patient also had elevated deoxyhemoglobin (HHb) not explained by methemoglobinemia alone; the low pulse oximetry (SpO_2_) reading suggested a rightward-shift oxyhemoglobin (O_2_Hb) dissociation curve, which is the opposite of that expected in methemoglobinemia. This, along with evidence of hemolysis, raised the possibility of a concomitant low-oxygen affinity hemoglobinopathy, which could explain elevated HHb. Hemoglobin electrophoresis identified an abnormal hemoglobin variant which was categorized as heterozygous for unstable beta globin variant. The patient responded well to one dose of methylene blue, vitamin C, supportive oxygen (O_2_) therapy, and IV hydration and was discharged with a baseline of 50-60% SpO_2_ on room air. We are reporting this case along with a brief review of the medical literature.

## Introduction

Congenital methemoglobinemia is characterized by an increase in the serum concentration of methemoglobin (MetHb); this occurs either due to congenital changes in hemoglobin synthesis or functional deficiency in the enzyme which is responsible for reducing methemoglobin in normal erythrocytes [1-4]. Methemoglobin is a form of hemoglobin in which has had its heme iron altered - from the ferrous (Fe^2+^) to the ferric (Fe^3+^) state - through oxidation. The true incidence of congenital methemoglobinemia has not been properly identified as it is often not diagnosed. Notably, the pathophysiology of congenital methemoglobinemia is unrelated to acquired methemoglobinemia, which occurs in response to toxin exposure [4]. Congenital methemoglobinemia is caused by a mutation leading to an altered form of hemoglobin (Hemoglobin M) or the absence of a necessary enzyme. Hemoglobin M is caused by mutations in either the alpha or beta chains of hemoglobin that results from a substitution of tyrosine for histidine.

In 1845, Francois, a French physician, described a patient with enduring congenital cyanosis in the absence of any obvious cardiac or pulmonary dysfunction and in the 1940s, Gibson showed that in these patients, there is a diminution in the ability of the erythrocytes to reduce methemoglobin [1,2]. Patients with hemoglobin M disease who have the alpha chain variant can present at birth with diffuse, persistent, slate-gray cyanosis, with no symptoms and no evidence of cardiopulmonary disease [1,4]. However, diagnosis is often delayed because of its low incidence, and cyanosis may not be clinically apparent [4]. However, patients with the beta chain variants usually present in the latter half of infancy. Failure of 100% oxygen to correct cyanosis is very suggestive of congenital methemoglobinemia. Cyanosis is typically seen on the nose, cheeks, fingers, toes, and mucous membranes (typical acrocyanosis). Incidentally, there is no clubbing. In 1959, Scott and Griffith identified the enzyme responsible for reduction of methemoglobinemia (Meth) [5]. It is an autosomal recessive disorder where the majority of affected individuals are deficient in the enzyme cytochrome b5 reductase (Cyb5R) due to pathogenic variants in the CYB5R3 gene. There are four types reported [6,7]. Type I Meth is the most common subtype where nicotinamide adenine dinucleotide (NADH) cytochrome b5 reductase enzyme is lacking only in the red blood cells. Jaffe and Hultquist said, “These patients are really more blue than sick” [8]. In Type II Meth NADH, cytochrome b5 reductase is deficient in various tissues and mental retardation is caused by a reduced oxygen supply to the central nervous system. The characteristic element of type 2 is a persistent, progressive neurologic deterioration. This disease is associated with mental retardation, microcephaly, opisthotonos, athetoid movements, and generalized hypertonia [9,10]. Type II constitutes approximately 10% of all cases and typically leads to death within the first few years of life as the severity of disease is an immediate result of the global deficiency in NADH-cytochrome b5 reductase. Type III Meth with NADH cytochrome b5 deficiency is extremely rare with only one case reported; it affects the entire hematopoietic system, the RBC, WBC and platelets. Type IV Meth is very similar to type I.

Of note, compensatory elevation of hemoglobin concentration is observed in patients with recessive hereditary methemoglobinemia, because of the methemoglobinemia induced left shift in the oxyhemoglobin dissociation curve. This shift leads to impaired oxygen release to the tissue [4,7,8].

Unstable hemoglobin variants are inherited mutations affecting globin genes that arise in the setting of rare mutational events. Some mutations of globin genes decrease the solubility of the molecules causing hemoglobin precipitation intracellularly. However, the mechanisms that contribute to hemoglobin instability vary, with over 1200 Hb variants (mutation), autosomal dominant (AD) and limited to a single pedigree [9,10]. Unstable Hb should be suspected in any patient with unexplained hemolysis and the typical clinical manifestations may include varying degrees of hemolysis, hemolytic anemia, and manifestations of hemolysis such as gallstones, splenomegaly. Initial labs should include routine laboratory studies for hemolytic anemia and a review of the peripheral blood smear. Genetic testing is the most definitive means of establishing the diagnosis and characterizing the variant.

## Case presentation

A three-year-old female was transferred from the pediatric clinic for profound oxygen desaturation in the 20-30% range. The patient was febrile, normotensive, with heart rate between 110-120 beats/minute. Physical examination revealed central and perioral cyanosis with increased respiratory rate (30 breaths/minute) and sternal and subcostal retractions. Auscultation of the lungs revealed transmitted upper respiratory sounds. Pulse oximetry was in the 60% range, while venous blood gas showed a pH of 7.42, partial pressure of carbon dioxide (pCO_2_) of 23, and bicarbonate (HCO_3_) of 72 mmol/L.

Laboratory findings showed a hemoglobin of 9 g/dL, with an elevated lactate dehydrogenase (LDH) of 1284 IU/L. Chest X-ray was unremarkable, electrocardiography showed normal sinus rhythm and echocardiogram (ECG) revealed normal function with no structural heart disease. The patient received one dose of Methylene blue 1 mg/kg before being admitted to the pediatric intensive care unit (PICU). Past medical history was significant as she was born to consanguineous parents, had normal growth and development and had a history of cyanosis since infancy. She was hospitalized two years prior to this visit for low oxygen saturation (SpO_2_ in the 50-60s, Methemoglobin level 12) at another institution where she was diagnosed with congenital methemoglobinemia without glucose-6-phosphate dehydrogenase deficiency. She did not have genetic testing. She was discharged with an oxygen saturation in the 60s and did not follow up post discharge. After admission to our institution, co-oximetry was performed that revealed O_2_Hb 68%, MetHb 16%, and HHb 14%. She remained stable and was discharged home after three days. After one month, she was re-admitted to the hospital with profound desaturation in the 20s despite appearing well in the setting of a respiratory illness. She was discharged home with oxygen saturation in the 50-60s range with hematology follow-up. In the hematology clinic, saturations were in the 60s and this was presumed to be her baseline.

A month later she presented with a fever/cough and decreased oral intake. In the emergency department, her oxygen saturation was in the 20s, respiratory rate was 12-18/minute; she was not in respiratory distress, lungs were clear to auscultation and the chest X-ray was negative. Influenza A was positive, and she was re-admitted to the pediatric intensive care unit. The oxygen saturations ranged between 20 and 40. Despite this low pulse oximetry reading, the patient was well-appearing/playful and in no distress. The Venous blood gas showed a pH of 7.42, pCO_2_ of 39 and partial pressure of oxygen (pO_2_) of 144 (reportedly venous), and co-oximetry from the same sample showed O2Hb 55%, MetHb 15%, HHb 30%; arterial blood gas (ABG) on room air showed a pH of 7.42, pCO_2_ of 23 and pO_2_ of 72 (Table 1); however, the lab could not process co-oximetry test from the same sample and reported that the blood was “viscous” or “chocolate like.” On a prior admission, ABG while on high flow nasal cannula (HFNC) showed a pH of 7.37, pCO_2_ of 44 and pO_2_ of 109 with a co-oximetry of O_2_Hb 68%, MetHb 16%, HHb 14%. The bilirubin was not elevated and G6PD was normal. Hb electrophoresis showed HbA1 > 90% with a beta globin gene variant of 4% - exact type unknown, influenza A was positive and the patient was started on oseltamivir. Urinalysis showed nitrites and urine culture had > 100,000 *E. Coli* for which she was started on antibiotics. Vitamin C was started for chronic treatment of presumed methemoglobinemia.

**Table 1:** Patient’s lab results Hb: hemoglobin; HCO3: bicarbonate; LDH: lactate dehydrogenase; O2: oxygen; O2Hb: oxyhemoglobin; pCO2: partial pressure of carbon dioxide; pO2: partial pressure of oxygen; SpO2: oxygen saturation; WBC: white blood cells; RBC: red blood cells; HCT: hematocrit; MCV: mean corpuscular volume; MCH: mean corpuscular hemoglobin; MCHC: mean corpuscular hemoglobin concentration; MPV: mean platelet volume; RDW: red cell distribution width; CRP: C-reactive protein.

CBC - Hemogram	06/2020
WBC	8.60 (5-21 x 10^3^/uL)
RBC	3.46 (4.05-5.35 x 10^6^ ul)
HGB	9.0 (10-15.1 g/dl)
HCT	28.4 (34-40%)
MCV	82.2 (98-118 fL)
MCH, POC	26.1 (31-37 pg)
MCHC	31.8 (30-36 g/dl)
RED BLOOD CELL DISTRIBUTION	15.4 (12.6-14.9%)
MPV	8.8 (7.9-11 fL)
PLATELETS	276 (120-340 x 10^3^/uL)

GENERAL CHEMISTRY	06/2020
GLUCOSE	72 (50-90 mg/dl)
BLOOD UREA NITROGEN	17.0 (9-20 mg/dl)
CREATININE	0.51 (0.6-1.2 mg/dl)
SODIUM	137 (128-148 mEq/L)
POTASSIUM	5.8 (3.5-5.1 mEq/L)
CHLORIDE	106 (96-111 mEq/L)
CO2	23 (22-30 mEq/L)
CALCIUM	10.0 (8.4-10.5 mg/dl)
ANION GAP	8.00 (8-13 mEq/dl)
ANION GAP WITH K	13.80 (7-17 mol/L)
LD	1284 (313-618 IU/L)
LACTATE	1.60 (0.7-2.1 mmol/L)
CRP	1.60 (0.5-1.0 mg/dl)

PATIENT INFORMATION	06/2020
FIO2 MODE (% OR LPM)	%
ARTERIAL	
PH ARTERIAL (APH)	7.36 (7.38-7.42)
PCO2, ARTERIAL	38.8 (35-45 mmHg)
PO2, ARTERIAL	66.2 (80-110 mmHg)
HCO3, ARTERIAL	21.2 (22-26 mEq/L)
BASE EXCESS, ARTERIAL	-3.9 (-3.0-3.0 mml)
O2 SAT, ARTERIAL	92.4 (95-100 mmHg)
CALCIUM IONIZED	5.0 (4.5-5.3 mg/dl)

HGB ELECTROPHORESIS	06/2020
RBC	3.33 (3.9-5.50 x 10^6^/ul)
HGB	8.9 (11.5-14.0 g/dl)
HCT	30.4 (34.0-42.0%)
MCV	91.3 (73.0-87.0 fL)
MCH	26.7 (24.0-30.0 pg)
RDW	17.7 (11.0-15.0%)
HGB A1	91.4 (>96.0%)
HGB A1 QUANT	3.2 (1.8-3.5%)
HGB F QUANT	0.8 (<2.0%)
ABNORMAL HGB-1	PRESUMPTIVE BETA GLOBIN VARIANT = 4.6%

The patient was discharged with plans to have hematology, cardiology and genetics follow-up; a letter was given to the family to be presented during emergency/doctor visits regarding anomalous pulse oximetry to help direct testing/disposition decisions in the future. Other labs were significant for a hemoglobin of 9, LDH in the 1200s, peripheral smear suggestive of mild hemolysis, co-oximetry showed MetHb of 15% (below the level as compared to the previous admission and below the treatment threshold). Since there was the possibility of hemolysis, the patient was followed by the hematology team.

## Discussion

Cyanosis is a bluish discoloration of the skin that is a useful clinical sign which can notify the clinician of significant underlying pathology [11]. Important differentials that require prompt investigation include pathologies involving the heart, lungs, airway, and vasculature. In this case, an unremarkable chest X-ray, ECG, and echocardiography ruled out structural abnormalities. The next differential to consider would be a hemoglobinopathy. In the case of methemoglobinemia, it can either be acquired from exposure to chemicals or medications or it can be congenital. Congenital methemoglobinemia can be caused by either a structural mutation of hemoglobin (more common) or by the deficiency of cytochrome b5 reductase, the enzyme intricately involved in methemoglobin processing (less common) [12,13]. Under oxidative stress, hemoglobin (Fe2+) is oxidized to methemoglobin (Fe3+). Methemoglobin, which normally constitutes 1% of the total hemoglobin, cannot carry oxygen. Normally, there is a balance of conversion back to hemoglobin (Fe2+) through the use of cytochrome b5 reductase. Without this healthy balance, the oxygen dissociation curve consequently gets shifted to the left because, interestingly, the oxygen binding affinity of normal hemoglobin is increased in the presence of an abnormal increase of methemoglobin, resulting in a decreased delivery of oxygen to the tissues [12,13].

Both of these occurrences contribute to a decreased amount of oxygen delivered to tissues; if it is to such a severe degree, hypoxemia and lactic acidosis can result. Clinical presentations typically depend on the percentage of methemoglobinemia. Three to 15% methemoglobin will cause slight discoloration of the skin and a level over 15-20% will cause cyanosis. Levels over 25-50% cause headache, dyspnea, dizziness, syncope, weakness, confusion, palpitations, and/or chest pain; levels at 50-70% cause delirium, seizures, coma, profound acidosis and levels over 70% usually result in death. It is also important to note that the failure of 100% oxygen to correct cyanosis is highly suggestive of methemoglobinemia. Measurement of oxygen saturation through a pulse oximeter is flawed in the case of methemoglobinemia because the pulse oximeter passes two wavelengths of light (600 and 940 nm) through tissue whereas methemoglobin absorbs light at 660 and 940 nm. This will cause inaccuracies and will necessitate the use of co-oximetry instead [4,10,13]. Co-oximetry is the gold standard for diagnosing methemoglobinemia because it measures four different wavelengths of light: namely, the wavelengths for carboxyhemoglobin, oxyhemoglobin, deoxyhemoglobin, and sulfhemoglobin [13]. Once methemoglobinemia is diagnosed, treatment will be based on the severity of symptoms primarily rather than on the numerical value of methemoglobinemia. Methylene blue is the treatment of choice for severe acute methemoglobinemia; hyperbaric oxygen or exchange transfusions are also acceptable treatment options if pharmacologic intervention fails or is contraindicated [4,13]. As previously mentioned, methemoglobinemia typically induces a leftward shift in the oxygen-hemoglobin dissociation curve. In our patient, the elevated HHb (14%) was not explained by methemoglobinemia alone, and these results in comparison to pO_2_ suggest a rightward-shifted oxyhemoglobin dissociation curve, which is the opposite of that expected in methemoglobinemia. In addition, the elevation in LDH suggested hemolysis. This raised the clinical suspicion of a concomitant low oxygen affinity hemoglobinopathy which prompted a closer look at the hemoglobin electrophoresis. There was an abnormal hemoglobin variant that was presumptively categorized as heterozygous for beta globin variant, possibly unstable [4,13]. This could explain the elevated HHb, right-shift of the O_2_Hb curve, hemolysis and well-appearance despite low saturation; this suggests that this rare patient with methemoglobinemia also had a structural change due to amino acid substitutions in the hemoglobin molecule at positions close to the heme groups. The clinical significance of this combination with an unstable variant is that patients with this mutation can present with hemolysis varying in severity, autooxidation and methemoglobinemia, spuriously low pulse oximetry readings, and a shorter lifespan. An astute and resourceful clinical team is required to make a proper and timely diagnosis of a hemoglobin variant. While correct identification of hemoglobin variants can be difficult due to complexity of the pathology as well as limitation of specific resources, this case brings to light the importance of meticulous investigation in the identification of hemoglobin variants. Planned genetic testing was not done due to non-compliance with follow-up.

## Conclusions

In conclusion, congenital methemoglobinemia may cause an apparent alarmingly low oxygen saturation when measured with standard pulse oximetry. It is important to keep a record of a known methemoglobinemia patient’s baseline oxygen saturation so as to enable clinically sound management decisions. Furthermore, if a patient presents clinically with signs of methemoglobinemia as well as with signs of hemolysis, unstable hemoglobin variants must be considered.
